# Optimising a Functional Beverage from Quinoa and Cherimoya Mixtures Fermented with Water Kefir Grains

**DOI:** 10.3390/foods14203464

**Published:** 2025-10-10

**Authors:** Abigail E. Palacios-Castillo, Tatiana N. Campoverde-Quilca, Jimmy Núñez-Pérez, Jhomaira L. Burbano-García, Holger M. Pineda-Flores, Rosario C. Espín-Valladares, Santiago Zárate-Baca, José-Manuel Pais-Chanfrau

**Affiliations:** 1 School of Agro-Industrial Engineering, Universidad Técnica del Norte, Ibarra 100105, Imbabura, Ecuadorjnunez@utn.edu.ec (J.N.-P.); jlburbano@utn.edu.ec (J.L.B.-G.); hmpineda@utn.edu.ec (H.M.P.-F.); rcespin@utn.edu.ec (R.C.E.-V.); 2 School of Biotechnology Engineering, Universidad Técnica del Norte, Ibarra 100105, Imbabura, Ecuador; szarate@utn.edu.ec

**Keywords:** *Chenopodium quinoa* W., *Annona cherimola* Mill., mixture experiment optimisation, probiotics, functional foods

## Abstract

Functional beverages enhance the nutritional value of their ingredients by increasing the levels of bioactive components, such as probiotics. To achieve consumer acceptance, functional beverages must be both palatable and nutritious. This study investigates the fermentation of quinoa and cherimoya at two temperatures (25 °C and 32 °C) using water kefir grains. The aim was to create a fermented mix that is both balanced and appealing to consumers. The response variables measured were the concentrations of lactic acid bacteria (LAB) and yeasts (CFU mL^−1^), as well as the overall liking (OL). Ten semi-trained panellists evaluated them using a seven-point hedonic scale. All three developed models for LAB and yeast growth, and OL exhibited *R*^2^ values exceeding 0.8, indicating a strong model fit and simultaneous optimisation considering the three key responses. At a temperature of 25 °C, the mass fractions of the mixes containing quinoa puree (QP) and cherimoya juice (CJ) were 0.13 and 0.87, respectively. Under optimal conditions, the LAB and yeast increased by 4.2 and 4.4 log units, respectively. Moreover, a significant 62% increase in protein levels and a notable 82% decrease in ascorbic acid were observed after 48 h of fermentation, likely caused by the Maillard reaction.

## 1. Introduction

Functional foods provide consumers with specific health benefits beyond their nutritional value [1]. Some fermented beverages possess these characteristics, and their contributions to health could improve the well-being of older individuals with digestive issues or intolerances to specific food components.

Another alternative could be to explore mixtures for creating these fermented beverages from different foods, as the nutrients provided by the individual parts of the mix can be enhanced by the contributions of specific bioactive compounds (such as enzymes, antimicrobials, antioxidants, and prebiotics) that microorganisms can synthesise.

This approach is particularly relevant for fermented beverages made from water kefir grains [2,3,4,5,6,7,8]. The contribution of the consortium of microorganisms that cohabits within the grain of water kefir, in terms of probiotic microorganisms, prebiotics, and various types of proteins, is comprehensive and well-documented. This supports the findings of previous research [7,9,10,11].

Fermented beverages made with water kefir granules can be well-received by individuals following a vegan and/or vegetarian lifestyle [12], as well as by celiac or lactose-intolerant patients [13,14], to whom it can meet some of their nutritional needs. This understanding and catering to specific dietary needs make these beverages suitable for meeting particular nutritional needs.

Water kefir grains have a unique ability to ferment various infusions, as well as fruit and vegetable extracts. The microorganisms in kefir grains play a crucial role in fermentation, adding health-promoting substances to the beverages [15,16,17].

Water kefir grains, a complex symbiotic culture of bacteria and yeasts (SCOBY), serve as crucial microbial reservoirs for fermenting sucrose solutions into a functional beverage. Quantitatively, these grains typically harbour high concentrations of microorganisms, with lactic acid bacteria (LAB) generally ranging from 10^7^ to 10^8^ colony-forming units (CFU) per gram of grain, and yeasts present at 10^6^ to 10^7^ CFU per gram of grain [18,19]. This microbial density within the grains is notably higher, often 10 to 30 times more concentrated, than in the resulting fermented liquid, underscoring their role as the primary inoculum and a robust, self-sustaining ecosystem [20].

Some fruit juices have been used to produce fermented beverages based on water kefir grains [21,22]. The juices provide sugars and other nutrients to the consortium of microorganisms in the water kefir grain, partially modify the substrate composition, and produce beneficial substances such as organic acids and antioxidant agents [21,22].

Fermentation time for natural substrates is significantly influenced by the concentration of carbon sources, especially polysaccharides and reducing sugars. In this regard, several studies have documented fermentation durations of around 48 h when employing water kefir grains [21,23,24,25].

For these purposes, cherimoya (*Annona cherimola* Mill.) can be used individually or conveniently mixed with other plant-based derivatives, such as Andean pseudocereals, which can provide high-value proteins, resulting in fermented beverages of high nutritional value.

Quinoa (*Chenopodium quinoa* Willd.) is one such pseudo-cereal used [26]. Quinoa protein concentrate, extracted mainly with hexane, has also been used in place of cooked quinoa food [27]. Its fermentation has aimed to increase the digestibility of the proteins in quinoa [27].

Such research has potential implications for the food industry. It could lead to the development of more digestible protein sources. Although direct fermentation of cherimoya with water kefir grains has not been previously reported, its fermentation with lactic acid bacteria has been reported [28].

Cooking quinoa increases the digestibility of its native proteins [29,30]. However, a certain bitterness is perceived as being unpalatable for some consumers, presumably due to its tannin content [31,32]. This rationale led to the strategy of mixing it with cherimoya juice before fermenting it with water kefir grains. In this way, the quinoa proteins would be fermented by water kefir grains, and the vitamins and taste of the cherimoya juice would provide valuable nutrients and flavours to the mixture during fermentation, improving the sensory attributes of the fermented beverage.

Multi-objective optimisation, a key aspect of this research, aims to discover conditions that meet multiple response objectives simultaneously [33]. Literature provides some examples. In one study, researchers sought to simultaneously maximise the production of LAB and yeasts in the fermentation process using milk or water kefir grains and a mixture of strawberry juice and tree tomatoes [34]. Another study determined the optimal conditions for maximising the concentration of kefiran, an edible prebiotic polymer and beneficial microorganisms (LAB and yeast), when milk kefir grains ferment whey [35]. In both cases, optimising several responses enabled the identification of the values of the factors that would allow the desired experimental objectives to be met.

The overall desirability function, a mathematical function linked to individual responses, plays a crucial role in the optimisation process involving several responses [36]. This function allows the simultaneous consideration of multiple responses, rather than optimising each individually. Maximising it enables us to determine the optimal factor levels for experimental success.

The basic idea behind this method is to convert each predicted response into a dimensionless desirability value, which ranges from zero to one. Zero indicates an undesired overall response, while a value of one indicates an entirely desirable one. After that, the individual desirability values are combined using geometric averaging to make the overall desirability function. This way, any response that is outside of permissible bounds will have an overall desirability of zero. This mathematical construction is proper when there are conflicting responses, as it helps find compromise solutions that strike the optimal balance among all the considered criteria.

The objective of the present research is to find the optimum composition of the mixture of cherimoya juice (hereafter CJ) and quinoa puree (hereafter QP) at two temperatures, using a desirability function that maximises the lactic acid bacteria and yeast concentration while minimising the overall liking score (where lower scores indicate higher preference) of the fermented drink with water kefir granules from a mixture of CJ and QP through a mixing experiment combined with two temperature levels.

## 2. Materials and Methods

### 2.1. Raw Materials

Water kefir grains (WKG) were obtained from a local supplier (www.kefir.ec (1/09/2025)). The WKGs were maintained exclusively in a fresh brown sugar solution (approximately 5% (*w*/*v*), prepared by dissolving 50 g of brown sugar in 1 L of distilled water) at room temperature, with the solution changed every three days. In each experiment, as reported by others [37] 100 g of the culture medium was inoculated with 10% (*w*/*w*) of WKG. Before being inoculated, WKG was washed with abundant drinking water and filtered through a clean plastic sieve.

The cherimoya (*A. cherimola* M.) and the quinoa (*C. quinoa* W.) flour were purchased from the central market in the city of Ibarra in Ecuador, always going to the same salesperson, who assured those suppliers came from the cantons ’Antonio Ante’ (in Imbabura province)—the cherimoya and ’Guamote’ (in Chimborazo province, Ecuador)—the quinoa. Quinoa was organically cultivated and processed into quinoa flour at a food processing plant located in Quito.

Organically certified farms in the ‘Guamote’ canton, Chimborazo province, Ecuador, supplied the quinoa used in this study. Synthetic fertilisers and pesticides are typically banned in organic farming. The well-drained, loamy-sandy soils of the Andean highlands are ideal for growing quinoa. It grows well in an environment with daytime temperatures of 15–20 °C, chilly nights, and a distinct dry season. The processing plant, GranAndino, certifies the product as organic, ensuring it meets these cultivation criteria and is free from synthetic substances.

Subtropical Andean valleys in the ‘Antonio Ante’ canton, Imbabura Province, Ecuador, are home to cherimoya cultivation. Due to the emphasis on fresh consumption, conventional cherimoya cultivation in this region often uses organic matter amendments and biological pest control methods rather than synthetic chemicals. Market vendors did not provide specific fertiliser or pest control practices for the exact batches used. Cherimoya grows on deep, fertile, well-drained volcanic soil. During its vegetative and fruiting phases, it thrives in warm temperatures (18–25 °C), high humidity, and evenly distributed rainfall.

### 2.2. Sample Preparation

#### Cherimoya Juice (CJ) and Quinoa Puree (QP) Preparations

**Figure 1:**
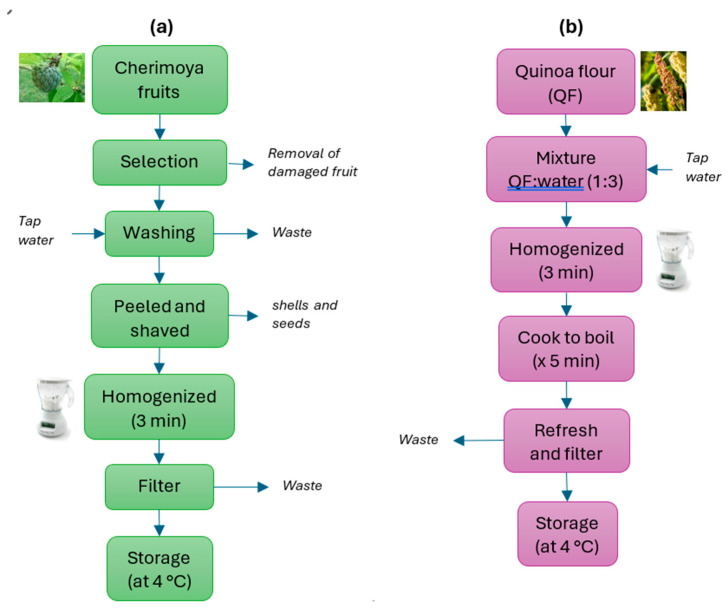
Block diagrams of the procedure followed to obtain (**a**) cherimoya juice and (**b**) quinoa puree used in the experiments.

The cherimoya was chosen at an advanced stage of maturity, as specified in the Ecuadorian technical standard NTE-INEN 2 475:2008. It was carefully selected to ensure it had no apparent external physical damage. It was then washed with plenty of tap water and left to dry for one hour. Then, the fruits were peeled, and the seeds were removed. The pulp of the cherimoya was inserted into a domestic blender for approximately 3 min, until a thick, white juice was obtained. This juice was then passed through a plastic sieve to remove the larger solids (Figure 1a).

The quinoa flour was mixed in a 1:3 ratio with still drinking water and homogenised for 3 min in a domestic blender. Subsequently, all the contents were cooked in an electric cooker until boiling, and then simmered for 5 min after boiling. Finally, after cooling, it was passed through a domestic sieve to remove the larger solids (Figure 1b).

Both CJ and QP were stored in a glass bottle with a hermetically sealed cap at 4 °C in a domestic refrigerator, and they should be used preferably within 24 h of preparation.

### 2.3. Measurements

#### 2.3.1. Enumeration of LAB and Yeast Colonies

Lactic acid bacteria and yeasts were counted using serial dilutions and then plated into disposable plate dishes using 3M™ Petrifilm™ Lactic Acid Bacteria Count Plate (3M Center, Building 275-5W-05 St. Paul, MN 55144-1000, USA) containing manufacturer-specific medium, and yeast potato-dextrose (YPD) (CompactDry™ YM) medium (Shimadzu Diagnostics Europe, 3 rue d’Alexandrie, 75002 Paris, France). The inoculated 3M™ Petrifilm™ plates were incubated at 28 °C for 48 h, and the CompactDry™ plates were incubated at 27 °C for 120 h. After incubation, 3M^TM^ Petrifilm^TM^ plates display red to reddish-brown LAB colonies, whereas CompactDry^TM^ YM plates display blue yeast colonies. In both cases, antifungal and antibiotic agents inhibit the growth of fungi and bacteria on 3M Petrifilm and CompactDry YM plates, respectively.

The enumeration methodology employed, which is predicated upon serial dilutions and plate counting, constitutes a standard and validated technique [38]. This approach facilitates the accurate quantification of microbial populations across substantial orders of magnitude, contingent upon the meticulous performance of appropriate dilutions to ensure the generation of discernible colonies.

The above seedings of each treatment were carried out at the start and end of the process, at 0 and 48 h, respectively. The *LAB* and *Yeast* responses were calculated as the difference between the end and the beginning of the fermentation process.

#### 2.3.2. Sensory Evaluation and Overall Liking Determination

The sensory variable comprises four weighted attributes of overall liking (*OL*): taste, smell, colour, and texture. Ten healthy volunteers, four males and six females, aged 18–40, were recruited and trained by the research team. They are neither smokers nor drinkers and have no prior experience in sensory analysis. The consumer panel used a seven-point hedonic scale to evaluate the final samples. The hedonic scale is similar to those employed in other studies [39,40].

The training consisted of an explanatory talk in which the panellists were informed about the objectives of the tests. Emphasis was placed on the individual nature of each panellist’s evaluations and how the assessment of any aspect of one of the samples prepared should not be shared or communicated to the rest afterwards.

Subsequently, after each panellist’s consent was approved and signed, a small test was conducted using a commercial beverage to verify their understanding of the task.

This scale evaluated each final sample’s taste, smell, colour, and texture attributes. It also evaluated its similarity to the raw materials used in producing the mixtures.

The hedonic scale (1 to 7) assigned the lowest values to the most favourable attributes and the highest values to the most unfavourable ones. Therefore, the optimisation aimed to find a combination of factors that minimises the *OL* score.

Overall likings (*OL*) were obtained by averaging the panellists’ ratings for each treatment.

#### 2.3.3. Ascorbic Acid Determination

The ascorbic acid (vitamin C) concentration in the samples was determined using the AOAC method 967.21/90 [41], which is suitable for maximum ascorbic acid concentrations of up to 50 ppm. A 2,6-dichloroindophenol at 400 ppm was used to titrate samples (5 g per treatment) diluted in 20 mL of a 2% oxalic acid solution. A standard solution of 0.2% ascorbic acid and a white solution corresponding to 0.2 mL of distilled water were used. The results were expressed in mg of vitamin C per 100 g of sample.

#### 2.3.4. Reducing Sugar and Total Protein Determination

The Luff–Schoorl method [42] was used to determine the free-reducing sugars, while the Kjeldahl method [43] determined the sample’s total protein content.

### 2.4. Experimental Design and Statistical Analysis

A combined mixture design was used, incorporating a two-level numerical factor (temperature at 25 °C and 32 °C) into the traditional simple two-blend lattice design. Design-Expert Software (version 23.1.1, 2024) (Stat-Ease, Inc., Minneapolis, MN, USA) was used to generate and analyse treatments randomly.

Each experimental unit used 250 mL bottles containing 200–220 mL of the CJ and QP mixture.

The lactic acid and yeast fermentations of the CJ and QP mixtures with water kefir grains were carried out for 48 h in an orbital incubator shaker (VS-8480SR, Bucheon-si, Gyeonggi-do, South Korea) with temperature control, according to the random order generated by Design-Expert^®^ statistical software used and according to the previously developed procedure [35].

The agitation speed was set at 80 rpm to maintain a homogeneous mixture, and the temperature was maintained at either 25 °C or 32 °C, as specified in the experimental design. The concentration of *LAB* and *Yeast* (CFU mL^−1^) and the pH were measured at the beginning and end of the process (see data in the Supplementary Material).

After running the experiments, the best models were generated for each response, seeking to be representative of the experimental values obtained and, subsequently, the combination of the factors was sought, with which the objectives of the functional beverage were achieved, maximising the content of *LAB* and *Yeast*, and the *OL* score was minimised.

The suitability of models is assessed by verifying the significance of the equation coefficients, aiming for a *p*-value of less than 0.05, through an analysis of variance (ANOVA).

The mathematical desirability function (D) approach is widely used in industry to optimise processes involving multiple response variables [44]. If a product or process has multiple quality features, the concept is that one will fall beyond the stated “desired” range [31]. The values of D fall between 0 and 1; if D=0, it is considered entirely undesirable, while D=1 represents the most desirable condition.

(1) $$D={\left({d_{LAB}}^{w_{LAB}}\cdot {d_{Yeast}}^{w_{Yeast}}\cdot {d_{OL}}^{w_{OL}}\right)}^{\frac{1}{\sum w_{i}}}$$

where $d_{LAB}$, $d_{Yeast}$, and $d_{OL}$ represent the individual desirability functions of each response, whereas $w_{LAB}$, $w_{Yeast}$, and $w_{OL}$ are the relative weights that these individual desires have in the overall desirable function (D).

The objective was to maximise the individual desirability functions $d_{LAB}$ and $d_{Yeast}$, while simultaneously minimising $d_{OL}$.

Functions $d_{LAB}$, $d_{Yeast}$ and $d_{OL}$ are determined as follows:

(2) $$d_{LAB}=\left\{\begin{array}{c} =0\;\;\;\;\;\;\;\;\;\;\;\;\;\;\;\;\;\;\;\;\;\;\;\;\;if\;{\hat{y}}_{LAB}<L_{LAB}\;\;\;\;\;\;\;\;\;\;\;\;\;\;\;\;\;\;\;\;\;\;\\ ={\left(\frac{{\hat{y}}_{LAB}-L_{LAB}}{T_{LAB}-L_{LAB}}\right)}^{s_{L}}\;if\;L_{LAB}\leq {\hat{y}}_{LAB}\leq T_{LAB}\;\;\;\;\;\;\;\;\;\;\;\;\;\;\\ =1\;\;\;\;\;\;\;\;\;\;\;\;\;\;\;\;\;\;\;\;\;\;\;\;\;if\;{\hat{y}}_{LAB}>T_{LAB}\;\;\;\;\;\;\;\;\;\;\;\;\;\;\;\;\;\;\;\;\; \end{array}\right.$$

(3) $$d_{Yeast}=\left\{\begin{array}{c} =0\;\;\;\;\;\;\;\;\;\;\;\;\;\;\;\;\;\;\;if\;{\hat{y}}_{Yeast}<L_{Yeast}\;\;\;\;\;\;\;\;\;\;\;\;\;\;\;\\ ={\left(\frac{{\hat{y}}_{Yeast}-L_{Yeast}}{T_{Yeast}-L_{Yeast}}\right)}^{s_{Y}}\;\;if\;L_{Yeast}\leq {\hat{y}}_{Yeast}\leq T_{Yeast}\;\;\;\;\;\;\\ =1\;\;\;\;\;\;\;\;\;\;\;\;\;\;\;\;\;\;\;if\;{\hat{y}}_{Yeast}>T_{Yeast}\;\;\;\;\;\;\;\;\;\;\;\;\;\;\; \end{array}\right.$$

(4) $$d_{OL}=\left\{\begin{array}{c} =1\;\;\;\;\;\;\;\;\;\;\;\;\;\;\;if\;{\hat{y}}_{OL}<T_{OL}\;\;\;\;\;\;\;\;\;\;\;\;\;\;\;\;\\ ={\left(\frac{{\hat{y}}_{OL}-U_{OL}}{T_{OL}-U_{OL}}\right)}^{s_{O}}\;\;if\;T_{OL}\leq {\hat{y}}_{OL}\leq U_{OL}\;\;\;\;\;\;\;\;\;\;\\ =0\;\;\;\;\;\;\;\;\;\;\;\;\;\;\;if\;{\hat{y}}_{OL}>U_{OL}\;\;\;\;\;\;\;\;\;\;\;\;\;\;\;\; \end{array}\right.$$

Note that individual functions $d_{LAB}$, $d_{Yeast}$, and $d_{OL}$, like D, always adopt values between 0 and 1.

The $L_{LAB}$ and $L_{Yeast}$ values are the lowest experimental values obtained from responses *LAB* and *Yeast*, respectively. In contrast, $T_{LAB}$ and $T_{Yeast}$ represent the highest experimentally achieved values from responses LAB and Yeast, respectively.

For the $d_{OL}$ response, the $T_{OL}$ and $U_{OL}$ The values represent the lower and higher experimental values of the *OL* response, respectively.

Finally, the coefficients $s_{L}$, $s_{Y}$, and $s_{O}$ represent the curvature of the desirability function as it approaches the desired value, being $s_{L}=s_{Y}=s_{O}=1$, the desirability function increases linearly to $T_{LAB}$, $T_{Yeast},$ or $T_{OL}$.

After verifying the suitability of the individual models, it proceeded to optimise D, with which the “optimum” values of the factors (CJ*, QP*, and temp*) and the highest D values can be obtained.

Accordingly, the data processing workflow follows the sequence: (Δ*LAB*, Δ*Yeast*, *OL*) → (${\hat{y}}_{LAB}$, ${\hat{y}}_{Yeast}$, ${\hat{y}}_{OL}$) → ($d_{LAB}$, $d_{Yeast}$, $d_{OL}$) → *D*. The ultimate objective is to identify the combination of factors (CJ, QP, Temp) that simultaneously maximise the models of *D*, ${\hat{y}}_{LAB}$, and ${\hat{y}}_{Yeast}$, while minimising ${\hat{y}}_{OL}$.

Conducting additional experiments under the best conditions (CJ*, QP*, and temp*) suggested by statistical optimisation provided further validation that the models were correct.

## 3. Results and Discussion

### 3.1. Multi-Response Optimisation for Mixed-Combined Experiments

As shown in Table 1, the experiments used the mixed-combined design of experiments (e.g., Kowalski, Cornell, Vining (KCV) model for combined designs) [45,46] to compare the proportions of CJ and QP in the mixture at two different temperatures (25 °C (−1.00) and 32 °C (+1.00)). The values shown are the growth rates of *LAB* and *Yeast* (CFU mL^−1^) and the average overall liking (*OL*) scores of 10 semi-trained panellists. The values of the responses from the different models studied are also reflected (Table 1).

The high concentrations of LAB and yeasts observed in our fermented beverage, consistently ranging from 10^7^ to 10^11^ CFU mL^−1^ (Table 1), are similar to the ranges reported in the scientific literature for various fermented products. For instance, Isas et al. (2020) documented LAB concentrations of 10^7^ to 10^9^ CFU mL^−1^ in fermented fruit juice [28], and Hecer et al. (2019) reported LAB yields as high as 10^5^ to 10^8^ CFU mL^−1^ in kefir-fermented milk [47]. These findings, coupled with the fact that commercial probiotic supplements often deliver 10^9^ to 10^10^ CFU per serving, underscore the technical feasibility and scientific basis for achieving such high microbial loads. The observed robust growth is further supported by the synergistic nutritional profile of the cherimoya and quinoa substrate, which provides both readily available simple sugars and a sustained release of complex carbohydrates, amino acids, and minerals, thereby creating an optimal environment for the proliferation of the water kefir microbiota.

The models obtained from each of the responses, in coded variable terms (e.g., *A* = coded proportion of CJ, *B* = coded proportion of QP, *C* = coded temperature), are the following:

(5) $${\frac{1}{\sqrt{{\hat{y}}_{LAB}-0.5}}=1.16\cdot 10}^{-4}\cdot CJ+1.28\cdot 10^{-4}\cdot QP+1.80\cdot 10^{-4}\cdot AB-3.1834\cdot 10^{-6}\cdot AC-3.9056\cdot 10^{-6}\cdot BC-5.5291\cdot 10^{-6}ABC\;\;\;\;\;\;\;\;\;\;\;\;\;\;\;\;\;\;\;\;\;\;\;\;\;\;\;$$

(6) $$\log_{10}\left({\hat{y}}_{Yeast}\right)=8.73\cdot A+9.96\cdot B+1.12\cdot C-3.51\cdot AB-1.27\cdot AC-1.77\cdot ABC\;\;+4.52\cdot AB\left(A-B\right)\;\;\;\;\;\;\;\;\;\;\;\;\;\;\;\;\;\;\;\;\;\;\;\;\;\;\;\;\;\;\;\;$$

(7) $${\hat{y}}_{OL}=3.12\cdot A+5.35\cdot B+0.14\cdot C\;\;\;\;\;\;\;\;\;\;\;\;\;\;\;\;\;\;\;\;\;\;\;\;\;\;\;\;\;\;\;\;\;\;\;\;\;\;\;\;\;\;\;\;\;\;\;\;\;\;$$

The response required transformation beforehand to represent the experimental values better when fitting the models of ${\hat{y}}_{LAB}$ (Equation (5)) and ${\hat{y}}_{Yeast}$ (Equation (6)).

The three models accurately predict the responses of the experimental runs, as attested by the analysis of variance and the goodness-of-fit statistics of each model (Table 2).

**Figure 2:**
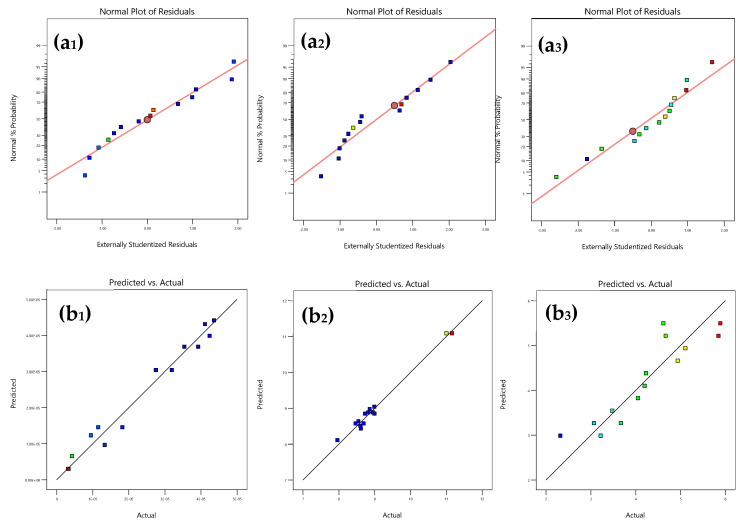
Quality analysis of the models obtained for the responses: *LAB* (**a_1_**,**b_1_**), *Yeast* (**a_2_**,**b_2_**) and *OL* (**a_3_**,**b_3_**). (**a_1_**–**a_3_**) normal plots of the residuals. (**b_1_**–**b_3_**) correspondence between the actual and model values of responses.

Additionally, the residuals of each model are normally distributed, and there is a good agreement between the actual and model-predicted values (see Table 1 and Figure 2).

As can be seen in the three models of the responses studied, ${\hat{y}}_{LAB}$ (Equation (5)), ${\hat{y}}_{Yeast}$ (Equation (6)), and ${\hat{y}}_{OL}$ (Equation (7)) are significant, with confidence levels > 99.9% (Table 2).

Crucially, the three models exhibit non-significant levels of lack of fit, indicating their suitability for accurately representing the experimental data (Table 2).

Finally, the models have *adequate precision* values greater than four, which allows them to identify extreme values where the responses are maximised or minimised (Table 2).

After confirming the models, the goal is to find a condition that maximises ${\hat{y}}_{LAB}$ and ${\hat{y}}_{Yeast}$ responses while minimising the ${\hat{y}}_{OL}$ score to the greatest extent possible. In the search for this condition, equal importance (weight = 3, see Table 3) was assigned to each of the three models obtained.

**Figure 3:**
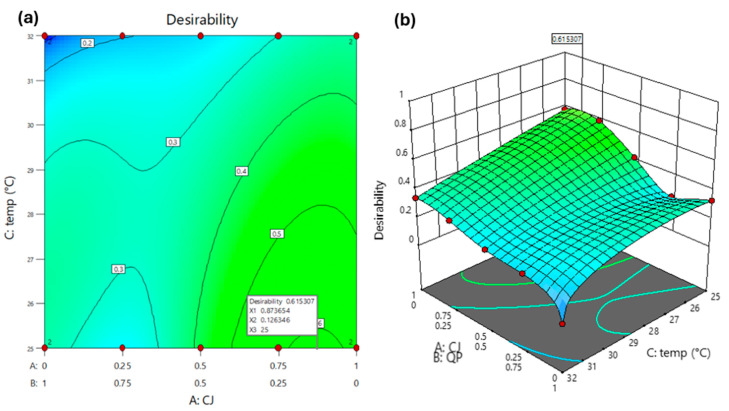
The overall function of desirability (*D*) is formed based on the models of the responses from *LAB, Yeast*, and *OL*. (**a**) Contour plot; (**b**) 3D plot.

The search performed under the conditions of Table 3 resulted in three optimums (Table 4), the first of which (CJ* = 0.87 (*w*/*w*); QP* = 0.13 (*w*/*w*); temp* = 25 °C) showed the highest value of the overall desirability function (D=0.62) (Figure 3). This point was chosen to conduct additional experiments and confirm the validity of the models.

The three optimal points yielded distinct qualitative interpretations of the overall desirability function values [31,40]. The first suggested optimum, with D=0.62, classifies as acceptably high (values of D between 0.6 and 0.8), while the second (D=0.58) classified as satisfactorily medium (between 0.4 and 0.6). The value for the third optimum (D=0.34) would be considered low (D-values between 0.2 and 0.4), falling between acceptable and unacceptable.

One of the three suggested optima, the first (No. 1), corresponds to a mixture of CJ and QP, whereas the other two optimal values correspond to points where only CJ (No. 2) or QP (No. 3) is used (Table 4).

The overall function of desirability (D) and its maximisation has been a mathematical tool widely used in multi-response optimisation [48,49]. Interpreting the combined desirability value can be challenging due to its complexity, and its physical interpretation may not be clear if the different objectives being combined in each response are not balanced and understood [50,51].

Numerous examples of its use can be found in industry. Its uses range from obtaining new alloys [52] to find the optimal semiconductor operating schedules [53]. It has also been used successfully in chemicals [54], and food [54,55].

Once the best solution (No. 1 in Table 4) was chosen, three more tests under the same conditions were suggested by the D function maximisation validating the model predictions (Table 5).

As shown in Table 5, the average and median values of all three responses to the confirmatory experiments fell within the ranges used in the models for each response (Table 6). This again shows that the previously developed models were accurate.

### 3.2. Comparison of the Optimal with the Raw Materials That Originate from Them

**Figure 4:**
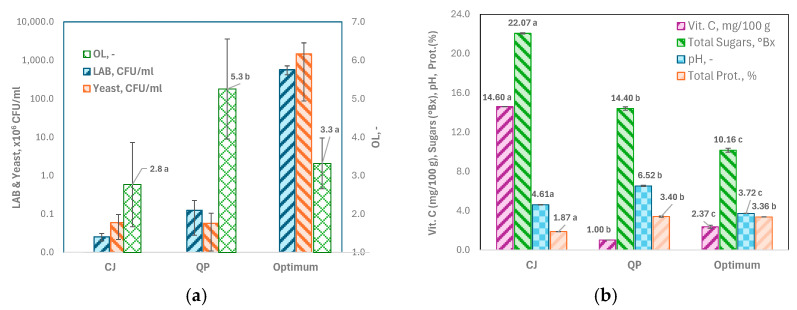
Comparison between the optimum obtained after 48 h fermentation with water kefir grains (Optimum) and the raw materials used (CJ: cherimoya juice, and QP: quinoa puree). (**a**) Probiotics (*LAB* and *Yeast*, in CFU mL^−1^) and overall liking score (*OL*); (**b**) Vitamin C (or ascorbic acid, mg/100 g), Sugars (° Brix), pH, and Total Proteins (%). Different letters denote statistically significant differences, as determined by the Tukey test (*p* < 0.05, *n* = 3).

The results show a notable increase in the presence of *LAB* and *Yeast*, with increases of 4.2 log units for *LAB* and 4.4 log units for *Yeast*. At the same time, *OL* values remain acceptably low compared to those in the raw material components (Figure 4a).

The contribution of CJ in the optimum offsets the bitterness of quinoa, as there is no significant difference (*p* > 0.05) between the *OL* scores of the CJ and the optimum (Figure 4a).

The favourable overall liking score, which showed no significant difference from the highly palatable cherimoya juice, indicates an effective mitigation of quinoa’s characteristic bitterness. This bitterness is primarily attributed to saponins located in the outer layers of the grain [31]. While common debittering strategies involve physical methods, such as washing or abrasion, or chemical treatments [32], this approach demonstrates a successful biological and formulation-based solution. The synergy likely operates on two levels: firstly, the inherent sweetness and creamy texture of cherimoya juice act as a powerful masking agent. Secondly, the metabolic activity of the water kefir consortium may have contributed to the enzymatic degradation of saponins, a phenomenon observed in other kefir-assisted fermentations, which has been shown to reduce saponin content and improve protein functionality [56]. This dual-action approach—masking and potential biological degradation—presents a natural and effective alternative to more intensive pre-processing methods for enhancing the consumer acceptability of quinoa-based products.

Furthermore, the optimum solution effectively preserves the levels of total proteins, primarily from quinoa, as there are no significant differences in this parameter between quinoa and the optimum (*p* > 0.05). Regarding vitamin C, while the initial levels from cherimoya are not matched, the optimum solution achieves values 1.4 times higher than those from quinoa (Figure 4b).

In addition, the mixture was 2.3 times more acidic under the optimum condition than the raw materials from which it originated (Figure 4b). The pH reduction could be associated with the metabolic activity of water kefir grain microbiota and the production of several organic acids, like lactic and acetic acids, as other researchers have reported [57,58].

The decline in ascorbic acid was noted earlier by other authors [59]. This is probably due to the Maillard reaction between reducing sugars in CJ and ascorbic acid. At the end of 48 h of fermentation, the reducing sugars in the optimum mixture reached 7.42 ± 1.11% (*w*/*w*) (*n* = 3) relative to the initial total sugars. Thus, the reducing sugars have most likely been present since the CJ was prepared, and therefore, since the beginning of the fermentation process.

The significant 82% reduction in ascorbic acid is a critical finding, though not unexpected in fermentation systems rich in both sugars and amino acids. While fermentation can sometimes preserve or even enhance vitamin levels, ascorbic acid is particularly susceptible to degradation. The proposed Maillard reaction pathway [59] is highly plausible in our system, given the abundance of reducing sugars from cherimoya and free amino groups from quinoa proteins, combined with a prolonged 48 h incubation period.

Similar losses have been reported in other fermented beverages and fruit-based products subjected to processing and storage [60]. Strategies to mitigate this loss often involve the addition of competitive chelating agents or other organic acids, such as citric acid, which can act as a sacrificial antioxidant to protect vitamin C [60,61]. This highlights that while our formulation is optimised for probiotic content and flavour, future iterations should focus on strategies to enhance the retention of sensitive micronutrients.

All the above suggest that the properties of the optimum support the claim of a functional beverage.

However, some strategies, including the addition of citric acid, can be implemented to counteract the degradation of ascorbic acid (vitamin C) [60]. It can substitute ascorbic acid in reacting with reducing sugars, minimising its degradation during fermentation and storage [61].

Other authors have focused on reducing quinoa’s bitterness, thereby improving its acceptability among consumers [32]. In this regard, various strategies have been proposed to reduce the saponin content in quinoa, which is attributed to bitterness. These include mixing flour with organic acids such as citric acid, heating and repeated washing with hot water, and using specific amounts of sodium bicarbonate [32].

Additional studies could be conducted to minimise ascorbic acid degradation in the fermented beverage and to determine the optimal conditions and storage time.

While this study primarily focused on optimising the fermentation process and characterising the fresh product, we acknowledge the critical importance of storage stability for the commercial viability and functional efficacy of the developed kefir beverage. Comprehensive long-term storage assessments, including the viability of LAB and yeasts and the evolution of sensory attributes, were beyond the scope of this initial research. However, preliminary insights from the literature indicate that water kefir beverages generally exhibit good microbial stability and a prolonged shelf life, particularly under refrigerated conditions (e.g., 4–5 °C). Studies have shown that viable LAB and yeast counts can be maintained at levels exceeding the minimum recommended for probiotic efficacy (typically 10^6^–10^7^ CFU mL^−1^) for periods of at least 30 days under refrigeration, while also preserving sensory qualities. Future research will specifically address these aspects, evaluating stability under various storage conditions (including room temperature and refrigeration) to determine the optimal shelf life and ensure consistent quality.

Other authors have obtained functional beverages based on cherimoya [28] and quinoa [56,62,63,64] separately; however, there are no reports on the fermentation of their mixtures.

The nutritional benefits observed in the fermented product are significantly attributable to the inherent composition of quinoa. Quinoa (*Chenopodium quinoa* Willd.) is renowned for its high content of biologically active compounds, which contribute to its beneficial health effects [65,66,67]. These include a rich array of polyphenols, flavonoids (such as quercetin and kaempferol), saponins, phytosterols, and essential amino acids, particularly lysine, which is often limited in other plant-based proteins [68]. These compounds collectively exhibit antioxidant, anti-inflammatory, and anticarcinogenic properties [69,70,71]. Furthermore, quinoa’s fibre content aids digestive health, while its complex carbohydrates provide sustained energy release [72,73]. The fermentation process can also enhance the bioavailability of some of these compounds by breaking down anti-nutritional factors and synthesising new beneficial metabolites [74,75,76].

Similarly, cherimoya (*Annona cherimola* Mill.) contributes significantly to the functional properties of the fermented beverage. This fruit is rich in various bioactive compounds, including phenolic compounds (such as proanthocyanidins and catechins), carotenoids, and a notable amount of ascorbic acid (Vitamin C), as well as B vitamins and dietary fibre [77,78,79,80]. The presence of these antioxidants helps combat oxidative stress in the body. Furthermore, cherimoya contains acetogenins, a unique class of compounds found in Annonaceae family, which have demonstrated potential antitumour and antiparasitic activities in various studies [80,81,82,83].

Although ascorbic acid levels decreased during fermentation in our study, the fruit’s overall contribution of other health-promoting compounds remains significant, complementing the nutritional profile derived from quinoa.

The remarkable increase in both LAB (4.2 log units) and yeast (4.4 log units) populations suggests a strong synergistic effect between cherimoya juice and quinoa purée as fermentation substrates, in combination with water kefir grains under the conditions presented herein. This phenomenon is not yet fully understood, and further research will be necessary to gain a deeper understanding of this observation.

While other studies using single fruit juices, such as mandarin or persimmon, have also reported successful fermentation by water kefir grains [21], the microbial density achieved in our research is exceptionally high. This can be attributed to the complementary nutritional profiles of the mixture. Cherimoya juice provides readily available simple sugars (e.g., fructose and glucose), fuelling the initial, rapid proliferation of the kefir microbiota [17]. Subsequently, the quinoa puree offers a sustained release of more complex carbohydrates, essential amino acids, and minerals, which are crucial for supporting the high cell densities observed at 48 h [27,84]. This dual-substrate strategy appears more effective than fermenting cherimoya alone, which primarily supports LAB growth up to approximately 10^8^ CFU mL^−1^ [28], a concentration several orders of magnitude lower than what we observed.

Qualitatively, the microbial community of water kefir grains exhibits significant diversity, with *Lacticaseibacillus paracasei, Lentilactobacillus hilgardii*, and *Liquorilactobacillus nagelii/satsumensis* frequently identified as dominant LAB species, while *Saccharomyces cerevisiae* is consistently the most prevalent yeast [15,85]. The precise composition is highly dynamic, influenced by factors such as geographical origin, cultivation conditions (e.g., temperature, fermentation time, water source), and the type and quantity of added substrates, including fruits or protein supplements [19,86]. This adaptability, along with the symbiotic metabolic cascade, where yeasts initiate sugar hydrolysis for lactobacilli and acetic acid bacteria (AAB), contributes to the grains’ resilience and the unique characteristics of the fermented product, including the production of dextran, which forms the grain’s physical structure.

In a study on the fermentation of cherimoya with five LAB strains, growth reaching approximately 10^8^ CFU mL^−1^ and a pH drop (-ΔpH) of roughly one unit were observed at the end of the fermentation process (48 h) and after storing the fermented product for 21 days at 4 °C [28]. The values of *LAB* concentrations in this study are 3–5 orders of magnitude lower than those obtained in the present research (see Table 1), likely due to the combined effect of a strong inoculum and the presence of quinoa.

The fermentation of quinoa has been studied more extensively [62,64]. Kefir grains have been able to ferment quinoa [64,84].

In one case, quinoa proteins were mixed with lentil flour proteins to improve solubility. After fermenting the mixture with water kefir grains, protein digestibility increased to 87%, the phenolic content also increased, and a decrease in saponin concentration was observed [84].

In the second case, milk kefir grains were used to ferment the quinoa protein concentrate at 37.4 °C for 12.4 h. Subsequently, different fruit and vegetable juices were added to enhance their sensory properties and to simulate in vitro gastrointestinal digestion [64].

The research team will conduct further studies to test these or other strategies for increasing consumer acceptance of nutritious and valuable mixes of Andean pseudocereal and fruits fermented with water kefir grains.

## 4. Conclusions

This study optimised a fermented beverage combining cherimoya juice (CJ) and quinoa purée (QP) through water kefir grain fermentation, employing a multi-response desirability function approach. The optimal formulation (0.87 CJ and 0.13 QP, *w*/*w*, at 25 °C) achieved a 4.2-log unit increase in lactic acid bacteria (LAB) and a 4.4-log unit increase in yeast concentrations, alongside a favourable overall liking score of 3.27 (on a seven-point scale where 1 = like extremely), demonstrating effective mitigation of quinoa’s bitterness through synergistic interactions with CJ. The blend retained 62% of total proteins but exhibited an 82% reduction in ascorbic acid, likely due to the Maillard reaction during fermentation, highlighting the need for strategies to preserve bioactive compounds.

The formulation offers a viable, plant-based, and probiotic-rich alternative for vegan, vegetarian, and lactose-intolerant consumers, aligning with global demands for sustainable and inclusive functional foods. Its methodological framework (integrating mixture design and desirability functions) provides a replicable model for optimising multifunctional fermented products from underutilised ingredients.

Future research should prioritise enhancing vitamin C retention through citric acid supplementation or modified fermentation conditions, alongside long-term stability assessments and large-scale sensory validation. Further exploration of Andean pseudocereals or fruits could expand product diversity, address nutritional and sensory challenges, while advancing regional agro-industrial valorisation. This study highlights the potential of traditional fermentation to convert culturally significant crops into innovative, health-promoting beverages.

Evaluating the shelf-life of the fermented product, specifically regarding the viability of lactic acid bacteria and yeasts, as well as the stability of its sensory attributes (taste, aroma, colour, and texture) under various storage conditions (e.g., refrigerated and ambient temperatures), is paramount for its potential commercialisation. Such stability studies would provide invaluable insights into the product’s longevity and guide appropriate packaging and distribution strategies, thereby constituting a logical and essential next step in this research endeavour.

## Figures and Tables

**Table 1:** Combined Mixture Design (KCV model) with the coded and the model-predicted response values. For *OL*, the experimental values are expressed as the mean ± standard deviation (*n* = 10). For LAB and yeast counts, they are reported as the median ± interquartile range (*n* = 3).

	Factors			Actual Responses					Model Responses		
Run	A: CJ (*w*/*w*) ^†^	B: QP (*w*/*w*)	C: Temp (°C)	*LAB*_0_ × 10^6^ CFU mL^−1^	Δ*LAB* × 10^9^ CFU mL^−1^	*Yeast*_0_ × 10^6^ CFU mL^−1^	Δ*Yeast* × 10^9^ CFU mL^−1^	*OL* Adim.	${\hat{y}}_{LAB}$ × 10^9^ CFU mL^−1^	${\hat{y}}_{Yeast}$ × 10^9^ CFU mL^−1^	${\hat{y}}_{OL}$ Adim.
1	0.25	0.75	−1.00	0.20 ± 0.02	0.56 ± 0.05	0.17 ± 0.01	0.10 ± 0.01	4.94 ± 0.40	0.63 ± 0.09	0.13 ± 0.00	4.65 ± 0.52
2	1.00	0.00	+1.00	2.43 ± 1.02	3.00 ± 0.00	0.30 ± 0.02	0.30 ± 0.06	3.67 ± 0.42	4.74 ± 0.64	0.38 ± 0.01	3.26 ± 0.37
3	0.50	0.50	+1.00	2.01 ± 0.67	5.60 ± 0.37	0.40 ± 0.02	0.40 ± 0.04	4.23 ± 0.54	10.8 ± 1.46	0.32 ± 0.01	4.38 ± 0.49
4	0.00	1.00	−1.00	0.16 ± 0.01	0.98 ± 0.07	0.10 ± 0.01	0.55 ± 0.04	5.85 ± 0.81	1.09 ± 0.15	0.70 ± 0.01	5.21 ± 0.59
5	0.25	0.75	+1.00	6.70 ± 0.45	55.0 ± 4.63	1.00 ± 0.00	0.99 ± 0.06	5.11 ± 0.87	23.6 ± 3.19	1.10 ± 0.02	4.93 ± 0.56
6	0.50	0.50	−1.00	0.19 ± 0.01	0.53 ± 0.06	0.12 ± 0.09	0.42 ± 0.05	4.20 ± 0.60	0.51 ± 0.07	0.27 ± 0.00	4.10 ± 0.46
7	0.75	0.25	−1.00	0.12 ± 0.00	0.59 ± 0.02	1.00 ± 0.08	0.73 ± 0.06	3.48 ± 0.38	0.54 ± 0.07	0.93 ± 0.02	3.54 ± 0.40
8	1.00	0.00	−1.00	0.41 ± 0.02	0.80 ± 0.05	0.10 ± 0.01	0.67 ± 0.09	3.22 ± 0.57	0.74 ± 0.10	0.76 ± 0.01	2.98 ± 0.34
9	0.75	0.25	+1.00	4.45 ± 1.67	4.45 ± 1.67	0.40 ± 0.04	0.36 ± 0.02	4.05 ± 0.36	6.63 ± 0.90	0.43 ± 0.01	3.82 ± 0.43
10	0.00	1.00	+1.00	2.80 ± 0.06	90.0 ± 2.93	1.45 ± 0.06	145 ± 7.07	5.89 ± 0.47	112 ± 15.1	120 ± 2.04	5.49 ± 0.62
11	0.00	1.00	−1.00	0.40 ± 0.03	1.32 ± 0.15	0.14 ± 0.01	1.00 ± 0.11	4.67 ± 0.77	1.09 ± 0.15	0.70 ± 0.01	5.21 ± 0.59
12	1.00	0.00	−1.00	0.58 ± 0.10	0.65 ± 0.02	0.31 ± 0.01	0.90 ± 0.00	2.32 ± 0.68	0.74 ± 0.10	0.75 ± 0.01	2.98 ± 0.34
13	0.00	1.00	+1.00	5.40 ± 1.84	9.00 ± 1.92	1.02 ± 0.05	102 ± 2.85	4.62 ± 0.83	11.2 ± 1.51	120 ± 2.04	5.49 ± 0.62
14	1.00	0.00	+1.00	3.20 ± 0.01	7.50 ± 0.88	0.50 ± 0.08	0.50 ± 0.08	3.07 ± 0.30	4.74 ± 0.64	0.38 ± 0.01	3.26 ± 0.37

^†^ (*w/w*) = weight/weight.

**Table 2:** Analysis of variance (ANOVA) of the *LAB*, *Yeast*, and *OL* regression models.

Source	Sum of Squares	df	Mean Square	*F*-Value	*p*-Value	
**Response 1:** ${\hat{y}}_{LAB}$**; Transform: inverse SQRT; constant: −0.5**						
**Model**	3.131 × 10^−9^	5	6.261 × 10^−10^	63.74	<0.0001	*significant*
Linear Mixture	1.824 × 10^−10^	1	1.824 × 10^−10^	18.56	0.0026	
*AB*	7.972 × 10^−11^	1	7.972 × 10^−11^	8.11	0.0215	
*AC*	5.298 × 10^−10^	1	5.298 × 10^−10^	53.93	<0.0001	
*BC*	7.975 × 10^−10^	1	7.975 × 10^−10^	81.18	<0.0001	
*ABC*	5.768 × 10^−11^	1	5.768 × 10^−11^	5.87	0.0417	
**Residual**	7.859 × 10^−11^	8	9.824 × 10^−12^			
*Lack of Fit*	3.896 × 10^−11^	4	9.739 × 10^−12^	0.9828	0.5065	*not significant*
*Pure Error*	3.964 × 10^−11^	4	9.909 × 10^−12^			
**Cor. Total**	3.209 × 10^−9^	13				
**Fit Statistics**						
Std. Dev.	3.134 × 10^−6^	*R*^2^ = 0.9755		*R*^2^-adj = 0.9602		
%C.V.	13.51	*Adeq. Precision:* 20.0314				
**Response 2:** ${\hat{y}}_{Yeast}$**; Transform: base 10 log; constant: 0**						
**Model**	10.6	6	1.77	74.61	<0.0001	*significant*
Linear Mixture	2.48	1	2.48	104.93	<0.0001	
*C*-temp	5.33	1	5.33	224.91	<0.0001	
*AB*	1.89	1	1.89	80	<0.0001	
*AC*	3.64	1	3.64	116.46	<0.0001	
*ABC*	0.485	1	0.485	20.48	0.0027	
*AB(A-B)*	0.6388	1	0.6388	26.98	0.0013	
**Residual**	0.1657	7	0.0237			
*Lack of Fit*	0.0868	3	0.0289	1.46	0.3506	*not significant*
*Pure Error*	0.079	4	0.0197			
**Cor. Total**	10.76	13				
**Fit Statistics**						
Std. Dev.	0.1539	*R*^2^ = 0.9846		*R*^2^-adj = 0.9714		
%C.V.	1.7	*Adeq. Precision:* 27.3387				
**Response 3:** ${\hat{y}}_{OL}$						
**Model**	11.41	2	5.7	24.91	<0.0001	*significant*
Linear Mixture	11.13	1	11.13	48.62	<0.0001	
*C*-temp	0.2744	1	0.2744	1.2	0.2971	
**Residual**	2.52	11	0.229			
*Lack of Fit*	0.4313	7	0.0616	0.1181	0.9919	*not significant*
*Pure Error*	2.09	4	0.5219			
**Cor. Total**	13.93	13				
**Fit Statistics**						
Std. Dev.	0.4785	*R*^2^ = 0.8191		*R*^2^-adj = 0.7862		
%C.V.	11.29	*Adeq. Precision:* 11.3058				

**Table 3:** The search criteria aim to find the conditions that maximise the LAB and yeast content increases while achieving the lowest possible OL values.

Name	Goal	Lower Limit	Upper Limit	Weight ^1^	Importance ^2^
*A*: CJ	is in range	0	1	1	3
*B*: QP	is in range	0	1	1	3
*C*: temp	is in range	25	32	1	3
${\hat{y}}_{LAB}$	maximise	5.25 × 10^8^	1.00 × 10^11^	1	3
${\hat{y}}_{Yeast}$	maximise	9.18 × 10^7^	1.45 × 10^11^	1	3
${\hat{y}}_{OL}$	minimise	2.3	5.9	1	3

^1^ $s_{L}=s_{Y}=s_{O}=1$; ^2^ $w_{LAB}=w_{Yeast}=w_{OL}=3$.

**Table 4:** Optimal values were given under the conditions shown in Table 3.

Number	CJ (*w*/*w*)	QP (*w*/*w*)	Temp (°C)	${\hat{y}}_{LAB}$ × 10^9^ CFU mL^−1^	${\hat{y}}_{Yeast}$ × 10^9^ CFU mL^−1^	${\hat{y}}_{OL}$ Adim.	Desirability Adim.
**1**	**0.87**	**0.13**	**25**	**0.62 ± 0.08**	**1.22 ± 0.02**	**3.27 ± 0.37**	**0.62**
2	1	0	25	0.75 ± 0.10	0.81 ± 0.01	2.98 ± 0.34	0.58
3	0	1	26.4	1.67 ± 0.23	2.03 ± 0.03	5.26 ± 0.59	0.34

**Table 5:** Confirmatory experiments for the validation of the models obtained.

Run	*LAB* × 10^9^ CFU mL^−1^	*Yeast* × 10^9^ CFU mL^−1^	*OL* Adim.
1	0.57	0.31	3.21
2	0.42	3	3.45
3	0.71	1.1	3.31

**Table 6:** Experiments confirm the values of each evaluated response under optimal conditions.

Response	Pred. Mean	Pred. Median	Std. Dev.	*n*	SE Pred	95% PI Low	Data Mean	95% PI High
*LAB*	6.1346 × 10^8^	6.0275 × 10^8^	9.3993 × 10^7^	3	N/A	4.6539 × 10^8^	5.4752 × 10^8^	8.1129 × 10^8^
*Yeast*	1.2181 × 10^9^	1.1440 × 10^9^	4.4548 × 10^8^	3	N/A	0.5526 × 10^9^	1.0076 × 10^9^	2.3684 × 10^9^
*OL*	3.2741	3.2741	0.4785	3	0.3507	2.5022	3.3233	4.046

## Data Availability

The original contributions presented in this study are included in the article/supplementary material. Further inquiries can be directed to the corresponding author.

## Supplementary Materials

The following supporting information can be downloaded at: https://www.mdpi.com/article/10.3390/foods14203464/s1, Files S1: manuscript-supplementary.xlsx.

## Abbreviations

The following abbreviations are used in this manuscript:

*A*	A code factor of CJ proportion
*B*	A code factor of QP proportion
*C*	A code factor of temperature
CJ	Mass fraction of cherimoya juice, *w*/*w*
*d_LAB_*, *d_Yeast_*, *d_OL_*	Desirability function of each response (*LAB, Yeast*, and *OL* score)
*D*	Overall desirability function
Δ*LAB*	Increment in lactic acid bacteria concentration, CFU mL^−1^
*LAB_0_*	Initial concentration of lactic acid bacteria, CFU mL^−1^
*OL*	Overall liking score
temp	Temperature of fermentation, °C
QP	Mass fraction of quinoa purée, *w*/*w*
Δ*Yeast*	Increment in yeast concentration, CFU mL^−1^
*Yeast*_0_	Initial concentration of yeast, CFU mL^−1^
${\hat{y}}_{LAB}$	Model to predict the increment in *LAB* concentration, CFU mL^−1^
${\hat{y}}_{OL}$	Model to predict the *OL* score
${\hat{y}}_{Yeast}$	Model to predict the increment in *Yeast* concentration, CFU mL^−1^
